# Different patterns of attentional bias in antenatal and postpartum depression

**DOI:** 10.1002/brb3.844

**Published:** 2017-10-18

**Authors:** Åsa Edvinsson, Alkistis Skalkidou, Charlotte Hellgren, Malin Gingnell, Lisa Ekselius, Mimmie Willebrand, Inger Sundström Poromaa

**Affiliations:** ^1^ Department of Women's and Children's Health Uppsala University Uppsala Sweden; ^2^ Department of Psychology Uppsala University Uppsala Sweden; ^3^ Department of Neuroscience, Psychiatry Uppsala University Uppsala Sweden

**Keywords:** antenatal depression, attentional bias, emotional Stroop, postpartum depression, pregnancy, women

## Abstract

**Background:**

Biased information processing in attention, memory, and interpretation is proposed to be central cognitive alterations in patients with major depressive disorder, but studies in women with peripartum depression are scarce. Because of the many similarities with depression in nonperipartum states as regards symptom profile and risk factors, we hypothesized that women with antenatal and postpartum depression would display attentional bias to negatively and positively valenced words.

**Methods:**

One hundred and seventy‐seven pregnant and 157 postpartum women were included. Among these, 40 suffered from antenatal depressive disorder and 33 from postpartum depressive disorder. An emotional Stroop task with neutral, positive, negative, and negatively valenced obstetric words was used.

**Results:**

No significant difference in emotional interference scores was noted between women with antenatal depression and nondepressed pregnant women. In contrast, women with postpartum depression displayed shorter reaction times to both positive (*p *=* *.028) and negative (*p *=* *.022) stimuli, compared with neutral words. Pregnant women on antidepressant treatment displayed longer reaction times to negatively valenced obstetric words in comparison with untreated depressed women (*p *=* *.012), and a trend toward greater interference in comparison with controls (*p *=* *.061).

**Conclusions:**

In contrast with the hypothesis, we found no evidence of attentional bias to emotionally valenced stimuli in women with untreated peripartum depression. However, the shorter reaction times to emotional stimuli in women with postpartum depression may indicate emotional numbing, which in turn, is a functional impairment that may have repercussions for child development and well‐being. Our findings emphasize the need to identify and treat women with postpartum depression at the earliest possible time point to ensure swift recovery and support for the family.

## 1 INTRODUCTION

Peripartum depression is a common but serious psychiatric disorder, with long‐lasting consequences for the mother, child, and family. The disorder is defined in the fifth edition of the *Diagnostic and Statistical Manual* of psychiatric disorders (*DSM–V*) as a depressive episode with onset during pregnancy or within 4 weeks of delivery (American Psychiatric Association, 2013). Irrespective of time of onset, that is, during pregnancy or after delivery, peripartum depression has substantial impact on women's lives in the critical first year after childbirth (Skalkidou, Hellgren, Comasco, Sylven, & Sundstrom Poromaa, 2012). The new *DSM–V* criteria have, however, been criticized with one of the concerns being the failure to incorporate distinct onset specifiers for pregnancy and the postpartum period, as such specifiers would stimulate and refine research on the etiology of the depressive episodes (Sharma & Mazmanian, 2014). As pregnancy and the postpartum period are characterized by profound differences in stress responsivity, sleep patterns, and endocrine milieu, the route by which depression develops and is maintained may differ (Skalkidou et al., 2012). Also, the consequences of depression during pregnancy and in the postpartum period are not the same. For instance, while antenatal depression may alter the fetal environment and result in low birth weight and preterm birth (Grote et al., 2010), the effects of depression in the postpartum period are commonly described in terms of impaired maternal–infant bonding. It is also important to notice that due to concerns about adverse effects from antidepressant use (Olivier et al., 2013), only about 25% of women with medicated pre‐pregnancy depression continue treatment during pregnancy, and individualized risk–benefit considerations are mandated (Ververs et al., 2006).

Biased information processing in attention, memory, and interpretation is proposed to be central cognitive alterations in patients with major depressive disorder (Gotlib & Joormann, 2010). According to cognitive theories, depression is caused and maintained by emotional processing bias and by deficits in cognitive control when negative information is processed, that is, depressed individuals selectively attend to negative information and/or have difficulty disengaging attention from negative stimuli (Beck, 2008; Gotlib & Joormann, 2010). Attentional bias to emotional information, one of the emotional processing biases encountered in depressed patients, can be studied by the emotional Stroop test (Williams, Mathews, & MacLeod, 1996). In this task, participants are asked to name the colors of words with different emotional contents while ignoring the meaning of the word. Attentional bias in this context is typified by longer latency (emotional interference) to name the color of affectively valenced words as compared with neutral ones. Attentional bias to negative stimuli has relatively consistently been reported in patients with depressive disorders (Cisler et al., 2011; Epp, Dobson, Dozois, & Frewen, 2012; Peckham, McHugh, & Otto, 2010), but seem to depend on the severity of the depressive episode, thus being more commonly found in patients with clinical depression and in patients with comorbid anxiety than in patients who merely present with depressive symptoms (Epp et al., 2012; Lyche, Jonassen, Stiles, Ulleberg, & Landro, 2011; Markela‐Lerenc et al., 2011). In addition, patients with depressive episodes may also present with attentional bias to positive stimuli in the emotional Stroop task, or lack of differentiation between the positive and negative stimuli (Epp et al., 2012; Gotlib & Joormann, 2010), or even with attentional bias away from emotional stimuli (Zvielli, Vrijsen, Koster, & Bernstein, 2016). A stronger and more consistent attentional bias toward threat‐related information has been described in patients with anxiety symptoms and disorders (Bar‐Haim, Lamy, Pergamin, Bakermans‐Kranenburg, & van IJzendoorn, 2007), and even in children with anxiety (Dudeney, Sharpe, & Hunt, 2015). It has thus been postulated that attentional biases toward threatening stimuli could be both a vulnerability factor as well as an important contributing factor to the maintenance of anxiety disorders (Heeren, Mogoase, Philippot, & McNally, 2015).

Biases in information processing appear to mediate the effects of genetic and environmental risk factors in the development of a depressive episode (Beck & Bredemeier, 2016). For instance, increasing evidence suggests that the short version of the serotonin transporter gene (*5‐HTTLPR*) is associated with attentional bias to negative stimuli (Pergamin‐Hight, Bakermans‐Kranenburg, van Ijzendoorn, & Bar‐Haim, 2012), and similar associations have been suggested for single nucleotide polymorphisms in the catechol‐O‐methyltransferase (*COMT*), dopamine receptor D2 (*DRD2*), and tryptophan hydroxylase (*TPH*) genes (Forssman et al., 2014; Gong et al., 2013). Similarly, childhood trauma and abuse predict attentional bias later in life (Gunther, Dannlowski, Kersting, & Suslow, 2015; Wingenfeld et al., 2011), and the interaction between stressful life events and genetic make‐up may further moderate the relationship (Jenness, Hankin, Young, & Smolen, 2016). Notably, some of the genes associated with attentional bias have also been implicated in peripartum depression (Comasco et al., 2011).

A substantial amount of research has examined the neural correlates of the attentional bias to negative stimuli. Negative attentional bias has been associated with impaired function and connectivity in top‐down attentional control, mediated by the dorsolateral prefrontal cortex and the dorsal anterior cingulate cortex (Banich, 2009; Beevers, Clasen, Stice, & Schnyer, 2010; Clasen, Beevers, Mumford, & Schnyer, 2014; Elliott, Rubinsztein, Sahakian, & Dolan, 2002; Eugene, Joormann, Cooney, Atlas, & Gotlib, 2010; Mitterschiffthaler et al., 2008; Silton et al., 2011; Szekely, Silton, Heller, Miller, & Mohanty, 2016). Furthermore, amygdala activity has been shown to correlate with attentional bias away from emotional stimuli in women with remitted depression (Albert, Gau, Taylor, & Newhouse, 2017).

Research along the lines of these cognitive theories is relatively scarce in antenatal and postpartum depression. As to antenatal depression, disrupted attentional processing of infant emotion has been reported in early pregnant women with depressive symptoms (Pearson, Cooper, Penton‐Voak, Lightman, & Evans, 2010). Similarly, increased selective attention to fearful faces has been described in distressed pregnant women, suggesting heightened sensitivity to danger cues during pregnancy (Roos et al., 2012). However, these studies have relied on subjective reporting of depressed mood and studies in women diagnosed with antenatal depressive disorder are lacking.

The great majority of postpartum depression studies has explored mother–infant interactions, which have been important for the understanding of short‐ and long‐term consequences for the offspring (Field, 2010). An extensive literature on maternal behavior suggests an essential role of the reward circuit in successful parenting, highlighting a set of hypothalamic–midbrain–limbic–paralimbic–cortical neural pathways important for human parental behaviors and feelings in response to infant cues (Nunes‐Costa, Figueiredo, & Moya‐Albiol, 2014; Swain, Perkins, Dayton, Finegood, & Ho, 2012). Postpartum depression is associated with impaired salience and fear networks activity as well as reduced corticolimbic responsiveness to infant‐related cues (Moses‐Kolko, Horner, Phillips, Hipwell, & Swain, 2014), and it is possible that difficulties in mother–child interaction may, in part, be due to negatively biased perceptions of the infant (Swain, Lorberbaum, Kose, & Strathearn, 2007). However, studies that have employed stimuli that are unrelated to motherhood are rare.

Furthermore, over the past years it has been debated whether antidepressant treatment is able to reverse the attentional bias to negative stimuli often noted in depressed patients. While some studies have demonstrated improved Stroop test performance during treatment (Wagner, Doering, Helmreich, Lieb, & Tadic, 2012), others have failed to demonstrate any treatment‐induced effects (Joormann & Gotlib, 2007; Nagane et al., 2014; Paelecke‐Habermann, Pohl, & Leplow, 2005; Teasdale & Dent, 1987), and findings remain controversial (Harmer, Cowen, & Goodwin, 2011). Even less is known about potential cognitive benefits of antidepressant treatment in peripartum depression, although such information would add value to the risk–benefit assessment of continued medication or initiation of treatment. In postpartum women, it seems that short‐term treatment with antidepressants has positive effects on appraisal of infant facial expressions, emphasizing the benefit of these drugs in the postpartum period (Stein et al., 2012). The situation in pregnant women is more complex, as clinical trials in this group are not feasible, and no randomized studies are available.

Due to the lack of studies using cognitive measures that would be valid for comparisons with nonpregnant or nonpostpartum populations, this study aimed at investigating attentional bias in women with antenatal and postpartum depressive disorders by use of the emotional Stroop task. Because of the many similarities with depression in nonperipartum states, as regards symptom profile and risk factors, we hypothesized that women with antenatal and postpartum depression would display attentional bias to negatively and positively valenced words in comparison with healthy controls. Furthermore, without a specific directional hypothesis, we aimed to investigate attentional bias in women who were on treatment with antidepressants for peripartum depression.

## 2 METHODS

### 2.1 Participants

In all, 201 pregnant and 173 postpartum women participated in this substudy to the pregnancy cohort “Biology, Affect, Stress, Imaging, and Cognition in pregnancy and the puerperium” (BASIC) between January 2010 and May 2013. The BASIC study is a longitudinal study investigating biological correlates of mood and anxiety disorders during pregnancy and in the postpartum period. All pregnant women in Uppsala County are invited to participate at the time of their routine ultrasound screening in gestational weeks 16–18 (Hannerfors et al., 2015; Hellgren, Akerud, Skalkidou, & Sundstrom‐Poromaa, 2013; Iliadis et al., 2015). Following informed consent, the women receive web‐based questionnaires, including the Swedish version of the Edinburgh Postnatal Depression Scale (EPDS) (Cox, Holden, & Sagovsky, 1987; Wickberg & Hwang, 1996) in gestational week 17, gestational week 32, 6 weeks postpartum, and 6 months postpartum. In gestational week 32, women report on current use of psychotropic medication during pregnancy.

Women with EPDS score ≥13 in gestational week 32 or current use of antidepressant therapy and a random sample of women with EPDS scores <13 in gestational week 32 were invited for a visit to the clinic with the intention of oversampling women with antenatal depressive symptoms (Rubertsson, Borjesson, Berglund, Josefsson, & Sydsjo, 2011). A similar procedure was undertaken for invitation of the postpartum women, where women with EPDS score ≥12 in postpartum week 6, and a random sample of women with EPDS scores <12 at the same time point were invited. A cut‐off point of 13 for depression during pregnancy (Rubertsson et al., 2011) and 12 for postpartum depression (Wickberg & Hwang, 1996) is often used for screening in clinical settings.

While EPDS scores were used to identify women *at risk* for peripartum depression, case status was defined based on psychiatric interview and ongoing use of antidepressants at the time of the evaluation. Exclusion criteria for pregnant women were serious pregnancy‐related complications (preeclampsia, intrauterine growth restriction, or gestational diabetes). Exclusion criteria for postpartum women were serious complications or disorders in the offspring requiring extended neonatal care. Pregnant women were assessed in gestational weeks 35–39 (according to the ultrasound‐estimated date of delivery) and postpartum women 6–14 weeks after delivery.

Women were interviewed about ongoing depressive disorders and primary anxiety disorders with the Swedish version of the Mini International Neuropsychiatric Interview (MINI) (Sheehan et al., 1998). Women with an ongoing minor (two to four symptoms persisting for at least 2 weeks) or an ongoing major depressive episode (at least five symptoms persisting for at least 2 weeks), or persistent depressive disorder (previously known as dysthymia) or ongoing use of antidepressants were considered to experience a depressive episode (*n* = 40 in pregnancy, and *n* = 33 postpartum). Women with comorbid anxiety were included (*n* = 19 in pregnancy, and *n* = 18 postpartum), but women who presented with anxiety‐only disorders (*n* = 24 in pregnancy, and *n* = 14 postpartum) were excluded as the focus in the study was on antenatal and postpartum depression.

In addition, the Montgomery–Åsberg Depression Rating Scale (MADRS‐S) and the EPDS were administered to assess depressive symptom severity (Cox et al., 1987; Montgomery & Asberg, 1979).

All participating women were interviewed about medical and obstetric history, alcohol use, smoking, breastfeeding status, and antidepressant treatment or other medication in the preceding 3 months. In addition, women were asked about total sleep duration (in hours) during the night preceding the test session. Sleep deprivation was defined as total sleep duration <4 hr. Data on height, first trimester weight, and visits to specialized care for fear of childbirth were obtained from the medical records. Fear of childbirth was defined as at least one visit made to the fear of childbirth clinic. Breastfeeding was categorized as no breastfeeding versus exclusive or partial breastfeeding.

All women provided written informed consent, and the study was approved by the Regional Ethical Review Board of Uppsala, Sweden, and the procedures were in accordance with the Helsinki Declaration of 1975 (revised in 2008).

### 2.2 Emotional Stroop task

Testing was carried out at the research laboratory at the Department of Women's and Children's Health, Uppsala University. The emotional Stroop task was designed using the E‐Prime psychology software tool (Psychology Software Tools, Inc., Sharpsburg, MD, USA) and contained 10 unique words from each word category: neutral, positive, negative, and negatively valenced obstetric words (Table S1). Each word was presented once in each color: blue, green, yellow, and red, resulting in a total of 160 word presentations, 40 of each category. The words were displayed against a black background on a 14‐inch laptop screen. Participants were asked to identify the color of the word while ignoring the meaning, and press the colored key that corresponded to the word color. Time to response was registered when the participant pressed the correctly colored keyboard letter, and the interstimulus interval was 2,000 milliseconds (ms). The test session was divided into two blocks with the possibility to take a short break half‐way through the session. Two postpartum women were excluded because they misunderstood the instructions.

The neutral, positive, and negative words were matched for number of syllables and linguistic frequency in Swedish. The negative words, part of which have been used previously (Sveen, Dyster‐Aas, & Willebrand, 2009; Willebrand et al., 2002), were selected in order to be emotionally relevant for the depressed participants. While many women suffered from comorbid anxiety, threatening words that would target anxiety were not included in the task. Instead, we included a set of negatively valenced obstetric words, which were matched against the other word categories for number of syllables. The obstetric words had lower linguistic frequency than the other word categories, but in this population of pregnant and postpartum women, we considered them to be more familiar than in the general population. The obstetric words were chosen based on low valence and high arousal to be comparable with the negative words. All words were validated in an independent sample of 40 pregnant women, by use of a self‐assessment Manikin scale ranging from 1 (*low valence, low arousal*) to 9 (*high valence, high arousal*) (Bradley & Lang, 1994). Valence for neutral, positive, negative, and obstetric words were 6.3 ± 0.7, 8.6 ± 0.3, 1.9 ± 0.6, and 3.1 ± 2.4, respectively. Arousal for neutral, positive, negative, and obstetric words were 2.7 ± 0.5, 3.2 ± 0.5, 5.6 ± 0.9, and 6.0 ± 1.4, respectively. Mean word length for neutral, positive, negative, and obstetric words were 6.6 ± 1.4, 7.3 ± 1.6, 7.0 ± 2.3, and 7.5 ± 2.0 letters, and corresponding number of syllables were 2.5 ± 0.7, 2.3 ± 0.7, 2.5 ± 0.5, and 2.5 ± 0.5, respectively.

### 2.3 Statistics

The power analysis was based on Pearson et al. (2013), and was targeted toward finding significant differences between women with depression and controls. According to the study by Pearson, where a mean difference between groups of 22 ms and an average standard deviation of 56 ms were reported, we assumed to detect an effect size of η^2^ = .28 (corresponding to a Cohen's *d *=* *.63). With one within‐group factor (three repeated measures) and two between‐group factors, the study had >80% power to detect a within–between interaction at an α‐level of .05 and a sample size of eight subjects per group. Demographic data were compared between groups by independent *t*‐tests or chi‐square tests.

Incorrect answers in the Stroop task were excluded (overall error rate was 0.5%). In addition, the first five responses of each individual were considered learning trials and were excluded. Also, responses with latencies greater than 2,000 ms (Stein et al., 2012) (<0.4%, equally spread across word categories) were excluded. For each individual participant, emotional interference scores were defined as reaction time for affectively valenced words minus reaction time for neutral words, and presented in ms.

For each group, normal distribution of emotional interference scores was ensured by the Shapiro–Wilks test. The overall emotional Stroop effect was evaluated by one‐way repeated measures ANOVA in the entire sample with word category (neutral, positive, negative, negatively valenced obstetric words) as within‐group variable, with the differences from neutral words evaluated by a simple contrast.

Thereafter, the emotional interference scores were modeled in a five‐way repeated measures ANOVA with word category (positive, negative, negatively valenced obstetric words) as within‐group variable, and perinatal state (pregnant vs. postpartum), depression (women with depression vs. nondepressed women), anxiety (women with anxiety vs. women without anxiety), and antidepressant use (use vs. nonuse) as between‐group variables. Age was included as a covariate in these analyses, as women with antenatal depressive disorder were significantly younger. To avoid spurious findings, no interactions beyond three‐way interactions were considered.

Although no significant main effect or interaction with perinatal state was noted in the omnibus ANOVA, we proceeded to conduct subgroup analyses in the pregnant women and postpartum women, respectively. This decision was based on requests in the field of perinatal mental health to separate antenatal depression from postpartum depression in order to elucidate potential important distinct etiologies (Sharma & Mazmanian, 2014). In addition, the endocrine milieu, stress reactivity, and risk factor profile differ between women with antenatal depression and postpartum depression (Skalkidou et al., 2012). Thus, 2 three‐way ANOVAs, one in pregnancy and one in the postpartum period, were performed with word category as within‐group variable, and depression and antidepressant use as between‐group variables. Anxiety was dropped as a factor in the perinatal state subgroup analyses as no main effect or interaction was noted in the omnibus ANOVA. When any of these 2 three‐way ANOVAs yielded a significant group × word category interaction, the interaction was evaluated by post hoc independent and paired *t*‐tests *and* confirmed by Mann–Whitney *U* test, comparing the emotional interference scores between groups or within groups. In addition, separate two‐way ANOVAs were made to evaluate the effects of breastfeeding, fear of child birth, and sleep deprivation. When applicable, Greenhouse‐Geisser‐corrected degrees of freedom, *F* and *p* values are presented. Effect sizes are reported as eta‐squared (η^2^). Correlations were performed by Spearman's rank correlation as the self‐rated depression scores did not meet the assumption of normal distribution. Statistical analyses were performed by use of SPSS Statistics 20.0.

## 3 RESULTS

One hundred and seventy‐seven pregnant and 157 postpartum women were available for the analyses. Demographic data for the study group are displayed in Table 1. Women with antenatal depressive disorder were significantly younger, less educated, and more often had a pre‐pregnancy psychiatric history than the pregnant controls. Women with postpartum depressive disorder also more commonly reported a pre‐pregnancy psychiatric history, and they were less often breastfeeding in comparison with the postpartum controls. Comorbid anxiety disorders were common in women suffering from both antenatal and postpartum depression, present in approximately half of cases (Table 1).

**Table 1:** Demographic data and clinical variables of the study group

	Pregnant women			Postpartum women		
	Controls (*n* = 137)	Antenatal depressive disorder (*n* = 40)	*p* a	Controls (*n* = 124)	Postpartum depressive disorder (*n* = 33)	*p* a
Age, years	31.8 ± 4.1	29.1 ± 5.3	.001	32.0 ± 4.5	30.4 ± 4.9	.09
Pre‐pregnancy BMI, kg/m^2^	22.7 ± 3.5	23.5 ± 4.4	.4	22.4 ± 3.0	24.0 ± 4.6	.08
Married/cohabiting, *n* (%)	136 (99.3)	39 (96.4)	.4	122 (98.4)	33 (100)	.5
University education	117 (86.7)	24 (61.5)	.003	99 (80.5)	21 (65.6)	.3
Parity, *n* (%)						
No previous children	60 (43.8)	22 (55.0)	.3	61 (49.2)	15 (45.5)	.8
At least one child	77 (56.2)	18 (45.0)		63 (50.8)	18 (54.5)	
Current smoking, *n* (%)	3 (2.2)	3 (7.5)	.2	3 (2.4)	1 (3.0)	.9
Current alcohol use, *n* (%)	0	1 (2.5)	.07	35 (28.2)^b^	5 (15.2)	.2
Breastfeeding, *n* (%)				115 (92.7)	25 (75.8)	.01
Sleep duration, hours	7.0 ± 1.4	6.6 ± 1.7	.3	6.7 ± 1.2	6.3 ± 1.3	.2
Fear of childbirth, *n* (%)	6 (4.4)	3 (7.5)	.5	4 (4.1)	4 (16.7)	.03
Pre‐pregnancy psychiatric history, *n* (%)	34 (24.8)	27 (67.5)	.001	24 (19.4)	16 (48.5)	.001
Comorbid anxiety disorder, *n* (%)	0	19 (47.5)	.001	0	18 (54.5)	.001
Depressive symptoms in pregnancy^c^, *n* (%)				11 (8.9)	12 (38.7)	.01
Antidepressant therapy, *n* (%)	0	15 (37.5)	.001	0	8 (24.2)	.001
SSRI		14 (35.0)			5 (15.2)	
SNRI		0			2 (6.1)	
Lamotrigine		1 (2.5)			1 (0.9)	
EPDS, median (IQR)	4 (1–6)	11 (6–14)	.001	3 (1–6)	13 (8–16)	.001
MADRS, median (IQR)	6 (3–11)	15.5 (9.5–22)	.001	5 (2–8)	18 (11.25–22.75)	.001

BMI, body mass index; IQR, interquartile range; EPDS, Edinburgh Postnatal Depression Scale; MADRS, Montgomery Åsberg Depression Rating Scale; SSRI, selective serotonin reuptake inhibitor; SNRI, serotonin–noradrenaline reuptake inhibitor.

^a^Unless indicated by superscript letter, *p*‐value denotes difference to control. Independent *t*‐test, Mann–Whitney *U* test, chi‐square test, or Fisher's exact test.

^b^Significantly more common than in pregnant controls, *p *<* *.001, Fisher's exact test.

^c^EPDS score ≥13 in gestational week 32, frequencies reported in relation to available data.

Women who used antidepressant treatment during pregnancy (*n* = 15) had lower scores of self‐rated depression than untreated depressed women (median MADRS 8 [IQR 3–16] vs. 20 [IQR 15–22], *Z *=* *3.16, *p *=* *.002; median EPDS 5 [IQR 3.5–10.5] vs. 13 [IQR 9.25–14.75], *Z *=* *−2.81, *p *=* *.005). A similar pattern was noted among postpartum antidepressant users (*n* = 8) (median MADRS 3 [IQR 0.5–10.5] vs. 20 [IQR 16–23], *Z *=* *−3.03, *p *=* *.002; median EPDS 3 [IQR 1–13] vs. 14 [IQR 12–17], *Z *=* *−2.38 *p *=* *.017). Women on antidepressant treatment during pregnancy had higher scores on the EPDS (*Z *=* *−2.19, *p *=* *.028), but similar MADRS scores (*Z *=* *−1.63, *p *=* *.103) as the healthy pregnant controls. EPDS and MADRS scores did not differ between women on antidepressant treatment and healthy controls in the postpartum period (*Z *=* *−0.14, *p *=* *.889; *Z *=* *−0.51, *p *=* *.609, respectively).

### 3.1 Emotional Stroop task

Reaction times for neutral, positive, negative, and negatively valenced obstetric words in the entire sample were 848 ± 125 ms, 838 ± 123 ms, 844 ± 124 ms, and 861 ± 133 ms, respectively. No differences in the reaction times to neutral words were found between pregnant and postpartum women (854 ± 135 ms vs. 840 ± 113 ms, *t *=* *0.97, *df* = 332, *p *=* *.332). Similarly, reaction times to neutral words did not differ between women with antenatal depression and nondepressed controls (antenatal depression 834 ± 93 ms vs. nondepressed pregnant women 858 ± 142 ms, *t *=* *1.10, *df* = 175, *p *=* *.275), or between women with postpartum depression and nondepressed postpartum controls (postpartum depression 855 ± 114 ms vs. nondepressed postpartum women 838 ± 113 ms, *t *=* *−0.68, *df* = 155, *p *=* *.495).

The emotional word categories induced a significant interference in comparison with neutral words, *F*(2.85, 333) = 51.73, *p *<* *.0001. This difference was primarily driven by longer reaction times to negatively valenced obstetric words, *F*(1, 333) = 44.33, *p *<* *.001, η^2^ = .12, and shorter reaction times to positive words, *F*(1, 333) = 32.70, *p *<* *.0001, η^2^ = .09, compared with the neutral words. The reaction times to negative words, on the contrary, did not differ from the neutral words, *F*(1, 333) = 2.45, *p *=* *.118.

### 3.2 Emotional interference by perinatal state, depressive and anxiety disorder, and antidepressant treatment

The omnibus ANOVA did not detect any main effect or interaction by perinatal state; main effect of perinatal state, *F*(1, 322) = 0.88, *p *=* *.350; perinatal state × depression, *F*(1, 322) = 0.008, *p *=* *.930; perinatal state × anxiety, *F*(1, 322) = 0.002, *p *=* *.965; perinatal state × antidepressant treatment, *F*(1, 322) = 0.35, *p *=* *.555; perinatal state × emotional word category interaction, *F*(1.91, 614.5) = 0.97, *p *=* *.375; perinatal state × word category × depression, *F*(1.91, 614.5) = 0.11, *p *=* *.884; perinatal state × word category × anxiety, *F*(1.91, 614.5) = 0.05, *p *=* *.940; and perinatal state × word category × antidepressant treatment, *F*(1.91, 614.5) = 0.38, *p *=* *.676.

No main effects of depression, anxiety, or antidepressant treatment were noted in the omnibus ANOVA; main effect of depression, *F*(1, 322) = 0.47, *p *=* *.495; main effect of anxiety, *F*(1, 322) = 0.04, *p *=* *.837; and main effect of antidepressant treatment, *F*(1, 322) = 1.85, *p *=* *.175. However, a significant word category × depression interaction was noted, *F*(1.91, 614.5) = 4.68, *p *=* *.011, η^2^ = .014, and similarly, a significant word category × antidepressant treatment interaction, *F*(1.91, 614.5) = 4.83, *p *=* *.009, η^2^ = .015. No interaction between word category and anxiety was found, *F*(1.91, 614.5) = 0.21, *p *=* *.798.

Furthermore, parity, all women: *F*(1, 332) = 0.11, *p *=* *.738; fear of childbirth, pregnant women: *F*(1, 175) = 0.75, *p *=* *.387; breastfeeding, postpartum women: *F*(1, 155) = 0.39, *p *=* *.533; or sleep deprivation, all women: *F*(1, 332) = 1.46, *p *=* *.227, did not influence the emotional interference scores for any of the affectively valenced word categories (data not shown).

### 3.3 Emotional interference by depression and antidepressant treatment in pregnant and postpartum women

**Figure 1:**
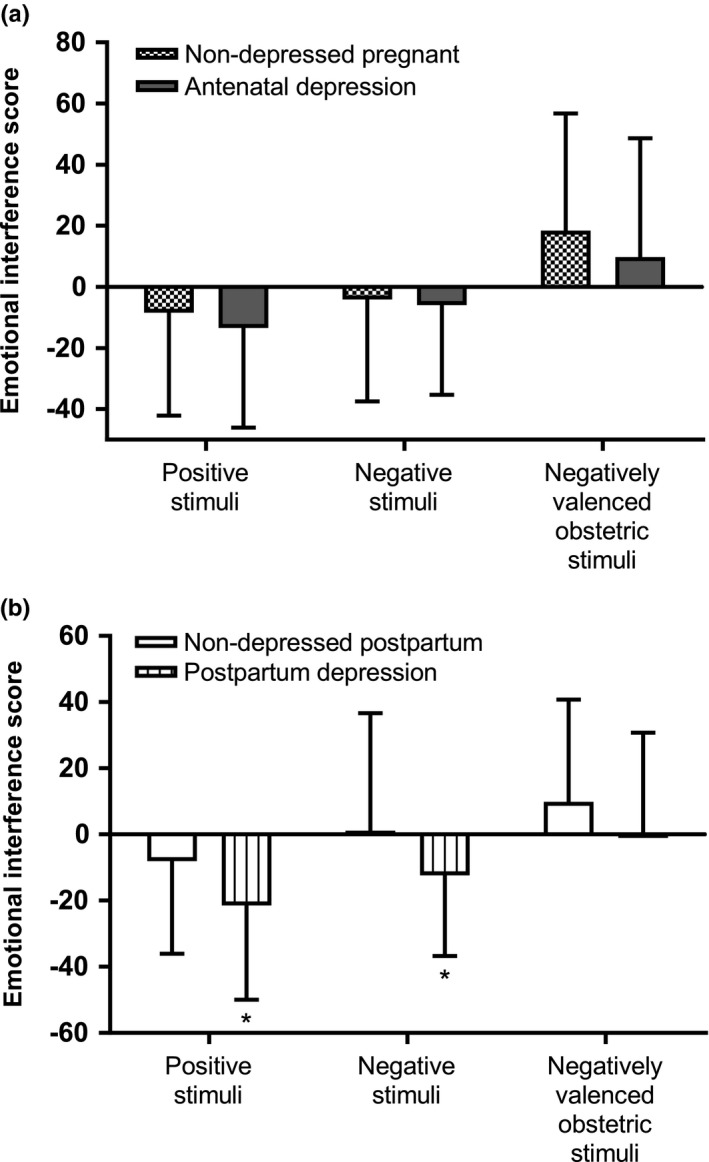
Emotional interference scores (mean ± SD) in (a) nondepressed pregnant women (*n* = 149) and women with antenatal depression (*n* = 28), and (b) nondepressed postpartum women (*n* = 131) and women with postpartum depression (*n* = 26). In this contrast, euthymic women on antidepressant treatment are regarded as nondepressed. No differences in emotional interference scores were noted between women with antenatal depression and nondepressed pregnant women. Women with postpartum depression displayed less emotional interference to positive and negative words, compared with nondepressed postpartum women (*p <* *.05, independent *t*‐test)

**Figure 2:**
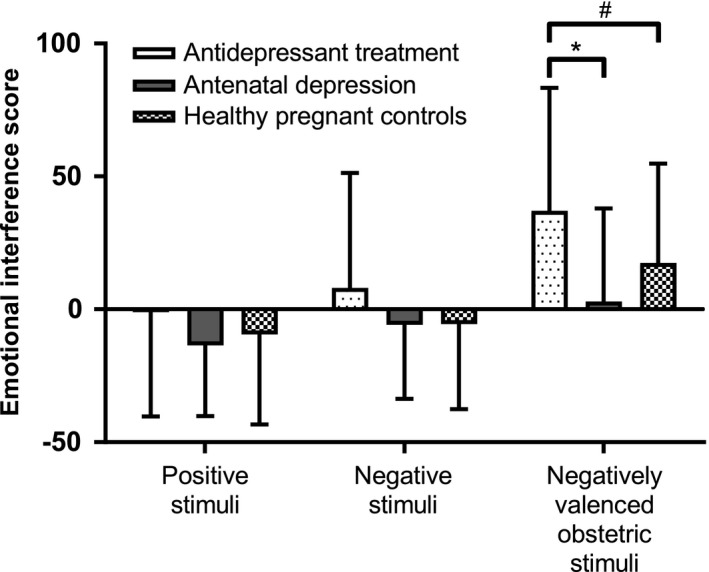
Emotional interference scores (mean ± SD) in pregnant women with antidepressant treatment (*n* = 15), nontreated depressed women (*n* = 25), and healthy controls (*n* = 137). Women on antidepressant treatment displayed greater emotional interference by negatively valenced obstetric stimuli than nontreated depressed women (*p =* *.012, independent *t*‐test), and a tendency to greater emotional interference by negatively valenced obstetric stimuli than healthy controls (^#^ *p *=* *.061, independent *t*‐test)

Two three‐way ANOVAs were used to elucidate the emotional interference in pregnant and postpartum women, respectively. In pregnancy, no significant difference in emotional interference scores was noted between depressed women and nondepressed women—main effect of depression, *F*(1, 172) = 0.36, *p *=* *.547; depression × emotional word category interaction, *F*(1.92, 330.7) = 2.44, *p *=* *.091 (Figure 1a). However, a trend toward a main effect of antidepressant use, *F*(1, 172) = 3.15, *p *=* *.078, and a significant interaction between antidepressant therapy and emotional word category, *F*(1.92, 330.7) = 4.34, *p *=* *.015, η^2^ = .03, were noted (Figure 2). Post hoc analyses of this interaction suggested that pregnant women on antidepressant therapy had a greater emotional interference by negatively valenced obstetric stimuli than untreated depressed pregnant women (mean difference = 34.2 ± 12.9 ms, *t *=* *2.65, *df* = 38, *p *=* *.012), and also a trend toward a greater interference in comparison with the healthy pregnant women (mean difference = 19.7 ± 10.4 ms, *t *=* *1.89, *df* = 150, *p *=* *.061) (Figure 2). No other significant post hoc findings were evident.

The three‐way ANOVA in postpartum women revealed no main effect of depression, *F*(1, 152) = 0.20, *p *=* *.659, or main effect of antidepressant treatment, *F*(1, 152) = 0.09, *p *=* *.764, but a significant depression × emotional word category interaction, *F*(1.83, 278.1) = 4.06, *p *=* *.021, η^2^ = .05. No antidepressant treatment × emotional word category interaction was noted, *F*(1.83, 278.1) = 1.40, *p *=* *.248. The interaction between postpartum depression and emotional word category was further evaluated by post hoc tests on the emotional interference scores in women with and without postpartum depression. According to the post hoc tests, women with ongoing postpartum depression displayed shorter reaction times to positive (mean difference = −13.4 ± 6.0 ms, *t *=* *−2.21, *df* = 155, *p *=* *.028) and negative (mean difference = −13.5 ± 5.7 ms, *t *=* *−2.37, *df = *155, *p *=* *.022) stimuli than to neutral words in comparison with the nondepressed women (Figure 1b). No other significant post hoc findings were evident.

In addition, the self‐rated MADRS and EPDS scores were significantly negatively correlated with the emotional interference scores by positive and negative stimuli, that is, with increasing MADRS or EPDS scores the emotional interference scores decreased (Table 2).

**Table 2:** Spearman's rank correlation coefficients for the correlations between emotional interference scores and self‐reported depression in pregnant and postpartum women

	Positive words Spearman's rho	Negative words Spearman's rho	Obstetric words Spearman's rho
Pregnancy			
MADRS	−.13	.05	.01
EPDS	−.07	.02	−.13
Postpartum			
MADRS	−.29***	−.20*	−.11
EPDS	−.25**	−.12	−.09

***p <* *.001, **p <* *.01, *p <* *.05, Spearman's rank correlation.

## 4 DISCUSSION

Women with untreated peripartum depressive disorder did not display attentional bias to affectively valenced stimuli. In contrast, women with postpartum depressive disorder had shorter reaction times to both positive and negatively valenced words. Only women with pre‐pregnancy depression who continued antidepressant treatment throughout pregnancy displayed the predicted attentional bias to negatively valenced obstetric words. No study has previously assessed attentional bias in women with antenatal or postpartum depressive disorder, why comparisons are limited to studies that have either used nonperipartum populations or studies using other emotional or cognitive paradigms.

The most important finding of the present study was that women with postpartum depression had shorter reaction times to both the positively and negatively valenced words than to the neutral words. In addition, depression severity, as measured by MADRS and EPDS scores, was significantly inversely correlated with the emotional interference scores. This finding can be interpreted in two ways; the shorter reaction times to the negative words may be the result of an explicit strategy to override the tendency for negatively valenced stimuli to interfere with color naming. This strategy has been noted previously in patients with chronic pain and in nonclinical high‐trait anxiety participants (de Ruiter & Brosschot, 1994; Puschmann & Sommer, 2011; Williams et al., 1996). It is an important coping strategy which may facilitate exit from the vicious circle of emotional processing bias and deficient cognitive control, which typifies the depressive episode (Williams et al., 1996). However, women with postpartum depression also displayed shorter reaction times to the positively valenced words, potentially suggesting they are emotionally numb to both negative and positive stimuli. This finding is in line with previous emotional and cognitive findings in women with postpartum depression. For instance, women with postpartum depression perform worse on emotion recognition tasks, where recognition of positive and negative emotional faces is impaired in comparison with healthy controls (Flanagan, White, & Carter, 2011). They are also *less* responsive to negative stimuli, with lower ratings of intensity and reactions to negative pictorial stimuli (Gollan, Hoxha, Getch, Sankin, & Michon, 2013), in contrast to the affective reactivity found in nonperipartum depressed women (Beck, 2008), and in contrast with the negatively biased interpretations of emotional stimuli found in healthy postpartum women (Hellgren, Bannbers, Akerud, Risbrough, & Poromaa, 2012). In addition, it has also been suggested that they tend to avoid or limit exposure to distressing infant stimuli (Field, 2010). Our findings are also in line with previous reports on disrupted attentional processing of infant emotion in pregnant women with depressive symptoms (Pearson et al., 2010).

If our findings are interpreted as signs of emotional numbing, they add to the long list of functional impairments that women with postpartum depression suffer from. Signs of emotional numbing at this important time in life may have long‐lasting consequences for child development and well‐being. Maternal sensitivity, healthy attachment, parental engagement, and reciprocal social interaction, all of which may be hampered in women with postpartum depression, are required for a normal psychoemotional development of the child (Alvarez, Meltzer‐Brody, Mandel, & Beeber, 2015). Our findings thus emphasize the need to identify and treat women with postpartum depression at the earliest possible time point to ensure swift recovery and support for the family. Cognitive behavioral therapy (CBT) is a well‐established and effective treatment for postpartum depression (Sockol, 2015), which fits well with the emotional and cognitive disturbances, noted by us and others, in these women. In addition, antidepressant treatment in the postpartum period, even as short term as 1 week, has positive effects on the depressive symptoms and on the appraisal of infant facial expressions (Stein et al., 2012). However, CBT treatment is less effective in antenatal depression than in postpartum depression (Sockol, 2015), and potentially this discrepancy may be explained by the absence of attentional bias noted among our untreated women with antenatal depression.

Relatively few studies in the area of peripartum depression have addressed neurobiological mechanisms that drive the development, or maintenance, of the depressive symptoms. Also from this perspective, the findings of this study represent a contribution to the field. It has been claimed that postpartum depression may have features that distinguish it from nonperipartum depression (O'Hara & McCabe, 2013). One of the major reasons for this assumption is the endocrine changes across pregnancy and the postpartum period, with pregnancy being characterized by supraphysiological hormone levels of estradiol, progesterone, cortisol, and corticotrophin‐releasing hormone, and the postpartum period instead typified by hormone withdrawal, low estradiol serum concentrations, low cortisol levels, and a lingering hyporesponsivity in the hypothalamic–pituitary–adrenal (HPA) axis (Skalkidou et al., 2012). While the exact hormonal changes that contribute to depression at this stage of reproductive life remains to be established, numerous hypotheses have been proposed (Skalkidou et al., 2012). Of relevance to the present results, HPA axis function, both at rest and in response to stress, seems to be associated with attentional bias. For instance, subjects with stress‐induced high cortisol levels display increased attentional bias toward depression‐related stimuli (Tsumura & Shimada, 2012) as well as positive stimuli (Dedovic et al., 2016). Thus, given the low cortisol levels that characterize the postpartum period, it is conceivable that the endocrine milieu also may have contributed to our finding. Finally, previous research has suggested that attentional bias is influenced by depression severity and comorbid anxiety (Cisler et al., 2011; Epp et al., 2012; Peckham et al., 2010). Greater effect sizes are noted in samples with clinical depression than in samples with depressed mood (Epp et al., 2012), and similarly, greater effect sizes are noted in cases with comorbid anxiety and depression than in cases who only suffer from depression (Epp et al., 2012). Although approximately 50% of participants had comorbid anxiety, we found no attentional bias to negative stimuli in peripartum depression. Furthermore, no main effect of comorbid anxiety disorder was noted. However, regarding the participants who suffered from comorbid anxiety, it should be stressed that the Stroop task employed in this study was not designed to specifically capture the attentional bias to threat, which characterizes anxiety disorders (Bar‐Haim et al., 2007). Clearly, our findings add to the growing literature suggesting that postpartum depression not only is characterized by time of onset, by demonstrating that these women do not display one of the cognitive alterations typical of depression, that is, attentional bias to negative stimuli. However, future head‐to‐head comparisons with a nonperipartum depressed control group are needed as null findings in nonperipartum depression also have been reported (Epp et al., 2012).

The other finding from our study was that women with pre‐pregnancy depression, who continued antidepressant treatment throughout pregnancy, displayed the expected attentional bias to negatively valenced stimuli. This finding may be interpreted as trait phenomenon (or scar from a previous depressive episode) or as being due to greater depression severity. Many studies have demonstrated that attentional bias to emotional content can be found in remitted patients, whether on antidepressant treatment or not (Joormann & Gotlib, 2007; Nagane et al., 2014; Paelecke‐Habermann et al., 2005; Teasdale & Dent, 1987), but findings are still mixed (Epp et al., 2012; Wagner et al., 2012). In line with the trait hypothesis, women on antidepressant treatment reported lower scores of self‐rated depression than the depressed women who managed without pharmacological treatment, suggesting the majority were in remission when tested. The other explanation is that these women have the greatest disease burden, that is, greater severity of depression. Most women with pre‐pregnancy depression or anxiety discontinue antidepressants when they plan a pregnancy or realize they are pregnant (Ververs et al., 2006). Only 25% of women with pre‐pregnancy antidepressant use continue treatment during pregnancy (Ververs et al., 2006), and these women are likely the ones in greatest need. This finding should also be interpreted with caution given the relatively low number of women who were using antidepressants during pregnancy, but if it is replicated, may add to the risk–benefit balance that must be addressed when advising pregnant women on whether to continue or discontinue treatment.

While it should be acknowledged that the relatively small sample of women with antenatal and postpartum depression limit the interpretation of the study results, a strength of the study is that diagnoses were based on structured psychiatric interview and not merely on presence of depressive symptoms, which is otherwise common in the field of peripartum depression. Also, given the direction of findings, it is unlikely that a greater sample size would have produced findings in line with our original hypothesis. Further limitations include the lack of a nonperipartum depressed control group, and the unmatched arousal ratings for positive and negative stimuli.

## 5 CONCLUSION

This study has demonstrated that women who suffer from antenatal and postpartum depression do not display the typical attentional bias to affectively valenced stimuli that is characteristic of depressive states in the nonpregnant population. Instead, women with postpartum depression displayed signs of emotional numbing, which may have repercussions for long‐term child development and well‐being. Our findings emphasize the need to identify and treat women with postpartum depression at the earliest possible time point to ensure swift recovery and support for the family.

## CONFLICT OF INTEREST

None declared.

## References

[brb3844-bib-0001] Albert, K. , Gau, V. , Taylor, W. D. , & Newhouse, P. A. (2017). Attention bias in older women with remitted depression is associated with enhanced amygdala activity and functional connectivity. Journal of Affective Disorders, 210, 49–56. 28012352 10.1016/j.jad.2016.12.010 PMC5292067

[brb3844-bib-0002] Alvarez, S. L. , Meltzer‐Brody, S. , Mandel, M. , & Beeber, L. (2015). Maternal depression and early intervention: A call for an integration of services. Infants and Young Children, 28(1), 72–87. 28316368 10.1097/IYC.0000000000000024 PMC5354305

[brb3844-bib-0003] American Psychiatric Association (2013). Diagnostic and statistical manual of mental disorders, fifth edition (DSM‐5). Arlington, VA: American Psychiatric Publishing.

[brb3844-bib-0004] Banich, M. T. (2009). Executive function: The search for an integrated account. Current Directions in Psychological Science, 18, 89–94.

[brb3844-bib-0005] Bar‐Haim, Y. , Lamy, D. , Pergamin, L. , Bakermans‐Kranenburg, M. J. , & van IJzendoorn, M. H. (2007). Threat‐related attentional bias in anxious and nonanxious individuals: A meta‐analytic study. Psychological Bulletin, 133(1), 1–24. 17201568 10.1037/0033-2909.133.1.1

[brb3844-bib-0006] Beck, A. T. (2008). The evolution of the cognitive model of depression and its neurobiological correlates. American Journal of Psychiatry, 165(8), 969–977. 18628348 10.1176/appi.ajp.2008.08050721

[brb3844-bib-0007] Beck, A. T. , & Bredemeier, K. (2016). A unified model of depression: Integrating clinical, cognitive, biological, and evolutionary perspectives. Clinical Psychological Science, 4(4), 596–619.

[brb3844-bib-0008] Beevers, C. G. , Clasen, P. , Stice, E. , & Schnyer, D. (2010). Depression symptoms and cognitive control of emotion cues: A functional magnetic resonance imaging study. Neuroscience, 167(1), 97–103. 20116416 10.1016/j.neuroscience.2010.01.047 PMC2840066

[brb3844-bib-0009] Bradley, M. M. , & Lang, P. J. (1994). Measuring emotion: The self‐assessment manikin and the semantic differential. Journal of Behavior Therapy and Experimental Psychiatry, 25(1), 49–59. 7962581 10.1016/0005-7916(94)90063-9

[brb3844-bib-0010] Cisler, J. M. , Wolitzky‐Taylor, K. B. , Adams, T. G. Jr , Babson, K. A. , Badour, C. L. , & Willems, J. L. (2011). The emotional Stroop task and posttraumatic stress disorder: A meta‐analysis. Clinical Psychology Review, 31(5), 817–828. 21545780 10.1016/j.cpr.2011.03.007 PMC3132173

[brb3844-bib-0011] Clasen, P. C. , Beevers, C. G. , Mumford, J. A. , & Schnyer, D. M. (2014). Cognitive control network connectivity in adolescent women with and without a parental history of depression. Developmental Cognitive Neuroscience, 7, 13–22. 24270043 10.1016/j.dcn.2013.10.008 PMC4209722

[brb3844-bib-0012] Comasco, E. , Sylven, S. M. , Papadopoulos, F. C. , Sundstrom‐Poromaa, I. , Oreland, L. , & Skalkidou, A. (2011). Postpartum depression symptoms: A case‐control study on monoaminergic functional polymorphisms and environmental stressors. Psychiatric Genetics, 21(1), 19–28. 21099450 10.1097/YPG.0b013e328341a3c1

[brb3844-bib-0013] Cox, J. L. , Holden, J. M. , & Sagovsky, R. (1987). Detection of postnatal depression. Development of the 10‐item Edinburgh Postnatal Depression Scale. British Journal of Psychiatry, 150, 782–786. 3651732 10.1192/bjp.150.6.782

[brb3844-bib-0014] de Ruiter, C. , & Brosschot, J. F. (1994). The emotional Stroop interference effect in anxiety: Attentional bias or cognitive avoidance? Behavior Research and Therapy, 32(3), 315–319. 10.1016/0005-7967(94)90128-7 8192630

[brb3844-bib-0015] Dedovic, K. , Giebl, S. , Duchesne, A. , Lue, S. D. , Andrews, J. , Efanov, S ., … Pruessner, J. C. (2016). Psychological, endocrine, and neural correlates of attentional bias in subclinical depression. Anxiety Stress and Coping, 29(5), 479–496. 10.1080/10615806.2015.1101457 26419271

[brb3844-bib-0016] Dudeney, J. , Sharpe, L. , & Hunt, C. (2015). Attentional bias towards threatening stimuli in children with anxiety: A meta‐analysis. Clinical Psychology Review, 40, 66–75. 26071667 10.1016/j.cpr.2015.05.007

[brb3844-bib-0017] Elliott, R. , Rubinsztein, J. S. , Sahakian, B. J. , & Dolan, R. J. (2002). The neural basis of mood‐congruent processing biases in depression. Archives of General Psychiatry, 59(7), 597–604. 12090812 10.1001/archpsyc.59.7.597

[brb3844-bib-0018] Epp, A. M. , Dobson, K. S. , Dozois, D. J. , & Frewen, P. A. (2012). A systematic meta‐analysis of the Stroop task in depression. Clinical Psychology Review, 32(4), 316–328. 22459792 10.1016/j.cpr.2012.02.005

[brb3844-bib-0019] Eugene, F. , Joormann, J. , Cooney, R. E. , Atlas, L. Y. , & Gotlib, I. H. (2010). Neural correlates of inhibitory deficits in depression. Psychiatry Research, 181(1), 30–35. 19962859 10.1016/j.pscychresns.2009.07.010 PMC2795107

[brb3844-bib-0020] Field, T. (2010). Postpartum depression effects on early interactions, parenting, and safety practices: A review. Infant Behavior and Development, 33(1), 1–6. 19962196 10.1016/j.infbeh.2009.10.005 PMC2819576

[brb3844-bib-0021] Flanagan, T. J. , White, H. , & Carter, B. G. (2011). Differential impairments in emotion face recognition in postpartum and nonpostpartum depressed women. Journal of Affective Disorders, 128(3), 314–318. 20800287 10.1016/j.jad.2010.07.021

[brb3844-bib-0022] Forssman, L. , Peltola, M. J. , Yrttiaho, S. , Puura, K. , Mononen, N. , Lehtimaki, T. , & Leppanen, J. M . (2014). Regulatory variant of the TPH2 gene and early life stress are associated with heightened attention to social signals of fear in infants. Journal of Child Psychology and Psychiatry, 55(7), 793–801. 24304270 10.1111/jcpp.12181

[brb3844-bib-0023] Gollan, J. K. , Hoxha, D. , Getch, S. , Sankin, L. , & Michon, R. (2013). Affective information processing in pregnancy and postpartum with and without major depression. Psychiatry Research, 206(2–3), 206–212. 23340374 10.1016/j.psychres.2012.11.030 PMC5697721

[brb3844-bib-0024] Gong, P. , Shen, G. , Li, S. , Zhang, G. , Fang, H. , Lei, L ., … Zhang, F. (2013). Genetic variations in COMT and DRD2 modulate attentional bias for affective facial expressions. PLoS One, 8(12), e81446. 24312552 10.1371/journal.pone.0081446 PMC3846795

[brb3844-bib-0025] Gotlib, I. H. , & Joormann, J. (2010). Cognition and depression: Current status and future directions. Annual Review of Clinical Psychology, 6, 285–312. 10.1146/annurev.clinpsy.121208.131305 PMC2845726 20192795

[brb3844-bib-0026] Grote, N. K. , Bridge, J. A. , Gavin, A. R. , Melville, J. L. , Iyengar, S. , & Katon, W. J. (2010). A meta‐analysis of depression during pregnancy and the risk of preterm birth, low birth weight, and intrauterine growth restriction. Archives of General Psychiatry, 67(10), 1012–1024. 20921117 10.1001/archgenpsychiatry.2010.111 PMC3025772

[brb3844-bib-0027] Gunther, V. , Dannlowski, U. , Kersting, A. , & Suslow, T. (2015). Associations between childhood maltreatment and emotion processing biases in major depression: Results from a dot‐probe task. BMC Psychiatry, 15, 123. 26047613 10.1186/s12888-015-0501-2 PMC4458030

[brb3844-bib-0028] Hannerfors, A. K. , Hellgren, C. , Schijven, D. , Iliadis, S. I. , Comasco, E. , Skalkidou, A ., … Sundstrom‐Poromaa, I. (2015). Treatment with serotonin reuptake inhibitors during pregnancy is associated with elevated corticotropin‐releasing hormone levels. Psychoneuroendocrinology, 58, 104–113. 25978816 10.1016/j.psyneuen.2015.04.009

[brb3844-bib-0029] Harmer, C. J. , Cowen, P. J. , & Goodwin, G. M. (2011). Efficacy markers in depression. Journal of Psychopharmacology, 25(9), 1148–1158. 20530590 10.1177/0269881110367722

[brb3844-bib-0030] Heeren, A. , Mogoase, C. , Philippot, P. , & McNally, R. J. (2015). Attention bias modification for social anxiety: A systematic review and meta‐analysis. Clinical Psychology Review, 40, 76–90. 26080314 10.1016/j.cpr.2015.06.001

[brb3844-bib-0031] Hellgren, C. , Akerud, H. , Skalkidou, A. , & Sundstrom‐Poromaa, I. (2013). Cortisol awakening response in late pregnancy in women with previous or ongoing depression. Psychoneuroendocrinology, 38(12), 3150–3154. 24041544 10.1016/j.psyneuen.2013.08.007

[brb3844-bib-0032] Hellgren, C. , Bannbers, E. , Akerud, H. , Risbrough, V. , & Poromaa, I. S. (2012). Decreased startle modulation during anticipation in the postpartum period in comparison to late pregnancy. Archives of Women's Mental Health, 15(2), 87–94. 10.1007/s00737-012-0261-7 PMC3305879 22315106

[brb3844-bib-0033] Iliadis, S. , Sylven, S. , Jocelien, O. , Hellgren, C. , Hannefors, A. K. , Elfstrom, D ., … Skalkidou, A. (2015). Corticotropin‐releasing hormone and postpartum depression: A longitudinal study. Psychoneuroendocrinology, 61, 61.

[brb3844-bib-0034] Jenness, J. L. , Hankin, B. L. , Young, J. F. , & Smolen, A. (2016). Stressful life events moderate the relationship between genes and biased attention to emotional faces in youth. Clinical Psychological Science, 4(3), 386–400. 27375963 10.1177/2167702615601000 PMC4928638

[brb3844-bib-0035] Joormann, J. , & Gotlib, I. H. (2007). Selective attention to emotional faces following recovery from depression. Journal of Abnormal Psychology, 116(1), 80–85. 17324018 10.1037/0021-843X.116.1.80

[brb3844-bib-0036] Lyche, P. , Jonassen, R. , Stiles, T. C. , Ulleberg, P. , & Landro, N. I. (2011). Attentional functions in major depressive disorders with and without comorbid anxiety. Archives of Clinical Neuropsychology, 26(1), 38–47. 21148667 10.1093/arclin/acq095

[brb3844-bib-0037] Markela‐Lerenc, J. , Kaiser, S. , Golz, T. , Fiedler, P. , Mundt, C. , & Weisbrod, M. (2011). Attentional bias in depressive patients and the moderating effect of concurrent anxiety. Psychopathology, 44(3), 193–200. 21412033 10.1159/000319370

[brb3844-bib-0038] Mitterschiffthaler, M. T. , Williams, S. C. , Walsh, N. D. , Cleare, A. J. , Donaldson, C. , Scott, J. , & Fu, C. H . (2008). Neural basis of the emotional Stroop interference effect in major depression. Psychological Medicine, 38(2), 247–256. 17825123 10.1017/S0033291707001523

[brb3844-bib-0039] Montgomery, S. A. , & Asberg, M. (1979). A new depression scale designed to be sensitive to change. British Journal of Psychiatry, 134, 382–389. 444788 10.1192/bjp.134.4.382

[brb3844-bib-0040] Moses‐Kolko, E. L. , Horner, M. S. , Phillips, M. L. , Hipwell, A. E. , & Swain, J. E. (2014). In search of neural endophenotypes of postpartum psychopathology and disrupted maternal caregiving. Journal of Neuroendocrinology, 26(10), 665–684. 25059408 10.1111/jne.12183 PMC4353923

[brb3844-bib-0041] Nagane, A. , Baba, H. , Nakano, Y. , Maeshima, H. , Hukatsu, M. , Ozawa, K ., … Arai, H . (2014). Comparative study of cognitive impairment between medicated and medication‐free patients with remitted major depression: Class‐specific influence by tricyclic antidepressants and newer antidepressants. Psychiatry Research, 218(1–2), 101–105. 24768252 10.1016/j.psychres.2014.04.013

[brb3844-bib-0042] Nunes‐Costa, R. A. , Figueiredo, B. , & Moya‐Albiol, L. (2014). The state of art of biological processes in paternal care. Psychology/Psicologia: Reflexão e Crítica, 4, 794–805.

[brb3844-bib-0043] O'Hara, M. W. , & McCabe, J. E. (2013). Postpartum depression: Current status and future directions. Annual Review of Clinical Psychology, 9, 379–407. 10.1146/annurev-clinpsy-050212-185612 23394227

[brb3844-bib-0044] Olivier, J. D. , Akerud, H. , Kaihola, H. , Pawluski, J. L. , Skalkidou, A. , Hogberg, U. , & Sundstrom‐Poromaa, I. (2013). The effects of maternal depression and maternal selective serotonin reuptake inhibitor exposure on offspring. Frontiers in Cellular Neuroscience, 7, 73. 23734100 10.3389/fncel.2013.00073 PMC3659337

[brb3844-bib-0045] Paelecke‐Habermann, Y. , Pohl, J. , & Leplow, B. (2005). Attention and executive functions in remitted major depression patients. Journal of Affective Disorders, 89(1–3), 125–135. 16324752 10.1016/j.jad.2005.09.006

[brb3844-bib-0046] Pearson, R. M. , Cooper, R. M. , Penton‐Voak, I. S. , Lightman, S. L. , & Evans, J. (2010). Depressive symptoms in early pregnancy disrupt attentional processing of infant emotion. Psychological Medicine, 40(4), 621–631. 19671214 10.1017/S0033291709990961

[brb3844-bib-0047] Pearson, R. M. , O'Mahen, H. , Burns, A. , Bennert, K. , Shepherd, C. , Baxter, H ., … Evans, J. (2013). The normalisation of disrupted attentional processing of infant distress in depressed pregnant women following Cognitive Behavioural Therapy. Journal of Affective Disorders, 145(2), 208–213. 22884235 10.1016/j.jad.2012.07.033

[brb3844-bib-0048] Peckham, A. D. , McHugh, R. K. , & Otto, M. W. (2010). A meta‐analysis of the magnitude of biased attention in depression. Depression of Anxiety, 27(12), 1135–1142. 21049527 10.1002/da.20755

[brb3844-bib-0049] Pergamin‐Hight, L. , Bakermans‐Kranenburg, M. J. , van Ijzendoorn, M. H. , & Bar‐Haim, Y. (2012). Variations in the promoter region of the serotonin transporter gene and biased attention for emotional information: A meta‐analysis. Biological Psychiatry, 71(4), 373–379. 22138391 10.1016/j.biopsych.2011.10.030

[brb3844-bib-0050] Puschmann, A. K. , & Sommer, C. (2011). Hypervigilance or avoidance of trigger related cues in migraineurs? – A case‐control study using the emotional Stroop task. BMC Neurology, 11, 141. 22054256 10.1186/1471-2377-11-141 PMC3235964

[brb3844-bib-0051] Roos, A. , Lochner, C. , Kidd, M. , van Honk, J. , Vythilingum, B. , & Stein, D. J. (2012). Selective attention to fearful faces during pregnancy. Progress in Neuro‐Psychopharmacology and Biological Psychiatry, 37(1), 76–80. 22154947 10.1016/j.pnpbp.2011.11.012

[brb3844-bib-0052] Rubertsson, C. , Borjesson, K. , Berglund, A. , Josefsson, A. , & Sydsjo, G. (2011). The Swedish validation of Edinburgh Postnatal Depression Scale (EPDS) during pregnancy. Nordic Journal of Psychiatry, 65(6), 414–418. 21728782 10.3109/08039488.2011.590606

[brb3844-bib-0053] Sharma, V. , & Mazmanian, D. (2014). The DSM‐5 peripartum specifier: Prospects and pitfalls. Archives of Women's Mental Health, 17(2), 171–173. 10.1007/s00737-013-0406-3 24414301

[brb3844-bib-0054] Sheehan, D. V. , Lecrubier, Y. , Sheehan, K. H. , Amorim, P. , Janavs, J. , Weiller, E. , … Dunbar, G. C. (1998). The Mini‐International Neuropsychiatric Interview (M.I.N.I.): The development and validation of a structured diagnostic psychiatric interview for DSM‐IV and ICD‐10. Journal of Clinical Psychiatry, 59(Suppl 20), 22–33; quiz 4–57. 9881538

[brb3844-bib-0055] Silton, R. L. , Heller, W. , Engels, A. S. , Towers, D. N. , Spielberg, J. M. , Edgar, J. C ., … Miller, G. A. (2011). Depression and anxious apprehension distinguish frontocingulate cortical activity during top‐down attentional control. Journal of Abnormal Psychology, 120(2), 272–285. 21553941 10.1037/a0023204 PMC4406398

[brb3844-bib-0056] Skalkidou, A. , Hellgren, C. , Comasco, E. , Sylven, S. , & Sundstrom Poromaa, I. (2012). Biological aspects of postpartum depression. Womens Health, 8(6), 659–672. 10.2217/whe.12.55 23181531

[brb3844-bib-0057] Sockol, L. E. (2015). A systematic review of the efficacy of cognitive behavioral therapy for treating and preventing perinatal depression. Journal of Affective Disorders, 177, 7–21. 25743368 10.1016/j.jad.2015.01.052

[brb3844-bib-0058] Stein, A. , Murphy, S. , Arteche, A. , Lehtonen, A. , Harvey, A. , Craske, M. G. , & Harmer, C. (2012). Effects of reboxetine and citalopram on appraisal of infant facial expressions and attentional biases. Journal of Psychopharmacology, 26(5), 670–676. 21948858 10.1177/0269881111421970 PMC3573684

[brb3844-bib-0059] Sveen, J. , Dyster‐Aas, J. , & Willebrand, M. (2009). Attentional bias and symptoms of posttraumatic stress disorder one year after burn injury. The Journal of Nervous and Mental Disease, 197(11), 850–855. 19996724 10.1097/NMD.0b013e3181bea555

[brb3844-bib-0060] Swain, J. E. , Lorberbaum, J. P. , Kose, S. , & Strathearn, L. (2007). Brain basis of early parent‐infant interactions: Psychology, physiology, and in vivo functional neuroimaging studies. Journal of Child Psychology and Psychiatry, 48(3–4), 262–287. 17355399 10.1111/j.1469-7610.2007.01731.x PMC4318551

[brb3844-bib-0061] Swain, J. E. , Perkins, S. C. , Dayton, C. J. , Finegood, E. D. , & Ho, S. S. (2012). Parental brain and socioeconomic epigenetic effects in human development. Behavioral and Brain Sciences, 35(5), 378–379. 23095400 10.1017/S0140525X12001112 PMC4317260

[brb3844-bib-0062] Szekely, A. , Silton, R. L. , Heller, W. , Miller, G. A. , & Mohanty, A. (2016). Differential functional connectivity of rostral anterior cingulate cortex during emotional interference. Social Cognitive and Affective Neuroscience, 12(3), 476–486. 10.1093/scan/nsw137 PMC5499751 27998997

[brb3844-bib-0063] Teasdale, J. D. , & Dent, J. (1987). Cognitive vulnerability to depression: An investigation of two hypotheses. British Journal of Clinical Psychology, 26(Pt 2), 113–126. 3580646 10.1111/j.2044-8260.1987.tb00737.x

[brb3844-bib-0064] Tsumura, H. , & Shimada, H. (2012). Acutely elevated cortisol in response to stressor is associated with attentional bias toward depression‐related stimuli but is not associated with attentional function. Applied Psychophysiology and Biofeedback, 37(1), 19–29. 21983835 10.1007/s10484-011-9172-z

[brb3844-bib-0065] Ververs, T. , Kaasenbrood, H. , Visser, G. , Schobben, F. , de Jong‐van den Berg, L. , & Egberts, T. (2006). Prevalence and patterns of antidepressant drug use during pregnancy. European Journal of Clinical Pharmacology, 62(10), 863–870. 16896784 10.1007/s00228-006-0177-0

[brb3844-bib-0066] Wagner, S. , Doering, B. , Helmreich, I. , Lieb, K. , & Tadic, A. (2012). A meta‐analysis of executive dysfunctions in unipolar major depressive disorder without psychotic symptoms and their changes during antidepressant treatment. Acta Psychiatrica Scandinavica, 125(4), 281–292. 22007857 10.1111/j.1600-0447.2011.01762.x

[brb3844-bib-0067] Wickberg, B. , & Hwang, C. P. (1996). The Edinburgh Postnatal Depression Scale: Validation on a Swedish community sample. Acta Psychiatrica Scandinavica, 94(3), 181–184. 8891084 10.1111/j.1600-0447.1996.tb09845.x

[brb3844-bib-0068] Willebrand, M. , Norlund, F. , Kildal, M. , Gerdin, B. , Ekselius, L. , & Andersson, G. (2002). Cognitive distortions in recovered burn patients: The emotional Stroop task and autobiographical memory test. Burns, 28(5), 465–471. 12163286 10.1016/s0305-4179(02)00052-9

[brb3844-bib-0069] Williams, J. M. , Mathews, A. , & MacLeod, C. (1996). The emotional Stroop task and psychopathology. Psychological Bulletin, 120(1), 3–24. 8711015 10.1037/0033-2909.120.1.3

[brb3844-bib-0070] Wingenfeld, K. , Riedesel, K. , Petrovic, Z. , Philippsen, C. , Meyer, B. , Rose, M ., … Spitzer, C. (2011). Impact of childhood trauma, alexithymia, dissociation, and emotion suppression on emotional Stroop task. Journal of Psychosomatic Research, 70(1), 53–58. 21193101 10.1016/j.jpsychores.2010.06.003

[brb3844-bib-0071] Zvielli, A. , Vrijsen, J. N. , Koster, E. H. , & Bernstein, A. (2016). Attentional bias temporal dynamics in remitted depression. Journal of Abnormal Psychology, 125(6), 768–776. 27505407 10.1037/abn0000190

