# Supplementary material for: Different patterns of attentional bias in antenatal and postpartum depression
Source: Brain Behav. 2017 Oct 18;7(11):e00844. doi: 10.1002/brb3.844 (PMC5698862; doi:10.1002/brb3.844)
Supplement: Supplementary file 1 [file BRB3-7-e00844-s001.docx]

**Supplementary table 1.** Emotional word categories for words used in the emotional Stroop task.

| **Neutral** | **Positive** | **Negative** | **Obstetric trauma** |
| --- | --- | --- | --- |
| Neutral | Fantastic | Apathy | Mastitis |
| Mountain | Prosperity | Depression | Stillbirth |
| Between | Freedom | Loss | Brain injury |
| Trade-union | Happy | Sad | Malformation |
| Tenant | Positive | Boredom | Miscarriage |
| Earphones | Gift | Failure | Unbearable |
| New moon | Success | Uneasy | Pain |
| Pie | Beautiful | Suicide | Laceration |
| Pilot | Winner | Grief | Vacuum extraction |
| Baker | Loves | Sleepless | Soreness |

A Swedish translation of the words can be obtained from the corresponding author.
